# The Prognostic Value of Phosphorylated AKT Expression in Non-Small Cell Lung Cancer: A Meta-Analysis

**DOI:** 10.1371/journal.pone.0081451

**Published:** 2013-12-05

**Authors:** Zhi-Xin Qiu, Kui Zhang, Xue-Song Qiu, Min Zhou, Wei-Min Li

**Affiliations:** 1 Department of Respiratory Medicine, West China Hospital, Sichuan University, Chengdu, P.R. China; 2 West China School of Preclinical and Forensic Medicine, Sichuan University, Chengdu, Sichuan, PR China; 3 Department of Pathology, Yubei District People’s Hospital, Chongqing, P.R. China; 4 Centers for Disease Control and Prevention, Changshou, Chongqing, P.R. China; H. Lee Moffitt Cancer Center & Research Institute, United States of America

## Abstract

**Objectives:**

Phosphorylated AKT (p-AKT), constitutive activation of AKT, is a potentially interesting prognostic marker and therapeutic target in non-small cell lung cancer (NSCLC). However, the available results of p-AKT expression in NSCLC are heterogeneous. Therefore, a meta-analysis of published researches investigating the prognostic relevance of p-AKT expression in patients with NSCLC was performed.

**Materials and Methods:**

A literature search via PubMed, EMBASE and CNKI (China National Knowledge Infrastructure) databases was conducted. Data from eligible studies were extracted and included into meta-analysis using a random effects model.

**Results:**

A total of 1049 patients from nine studies were included in the meta-analysis. Nine studies investigated the relationship between p-AKT expression and overall survival using univariate analysis, and five of these undertook multivariate analysis. The pooled hazard ratio (HR) for overall survival was 1.49 (95% confidence interval (CI): 1.01-2.20) by univariate analysis and 1.02 (95% CI: 0.54-1.95) by multivariate analysis.

**Conclusion:**

Our study shows that positive expression of p-AKT is associated with poor prognosis in patients with NSCLC. However, adequately designed prospective studies need to perform.

## Introduction

 Lung cancer is one of the most common human cancers and the leading cause of cancer-related deaths with non-small cell lung cancer (NSCLC) accounting for ~80% of all primary lung cancers. Despite advances in surgery, chemotherapy, and radiotherapy, the 5-year survival rate of patient with NSCLC is only 15% [1]. Thus, the lack of major improvements in the 5-year survival rate of NSCLC has driven the search for new strategies aimed at improving lung cancer management. Current knowledge regarding NSCLC is not a single disease but a collection of diseases with distinct pathogeneses by molecular mechanisms. Changes of genetic and epigenetic play an integral role in the transformation, promotion and progression of cancer. Therefore, understanding the biology of lung cancer and finding the molecular markers may useful for predicting survival and aiding the management of patients with NSCLC [2].

 Activation of the intracellular prosurvival signal transduction protein AKT, or protein kinase B, is a Serine/Threonine protein kinase, has been proposed as a central signaling event in carcinogenesis [3]. The role of AKT in carcinogenesis has been well documented and overexpressions of AKT are found in a variety of human cancer types [4,5]. Moreover, AKT has been associated with the initiation of tumorgenesis in gliomas [6] and pancreatic cancer [7] and seems to correlate with stage and tumor grade in prostate cancer [8]. Phosphorylated AKT (p-AKT) is the constitutive activation form of AKT that is a powerful promoter of cell survival as it antagonizes and inactivates various components of the apoptotic cascade such as caspase-9 [9], Bad [4], and forkhead transcription factor family members [10]. Moreover, AKT has been implicated in regulating angiogenesis [11] and metastasis [12], which are two important processes in tumor development.

 The effect of abnormalities expression of p-AKT has been investigated for NSCLC by using univariate or multivariate analysis, however, the prognostic significance of p-AKT expression on patients with NSCLC remains controversial. Based on the discordant results obtained by numbers of studies, we conducted this meta-analysis to quantify the prognostic impact of p-AKT expression on overall survival among patients with NSCLC.

## Materials and Methods

### Literature Search

 A literature search via PubMed, EMBASE and CNKI (China National Knowledge Infrastructure) databases was conducted to find articles that evaluated the role in NSCLC (Last search was updated on July 7, 2013). The keywords and text words were used as follows: (1) Phosphorylated AKT or AKT phosphorylation or phosphor-AKT or p-AKT, and (2) non-small cell lung cancer or NSCLC, and (3) prognostic or survival analysis or expression.

### Selection Criteria

 All languages were included, and all eligible articles that examined the association between the expression of p-AKT and overall survival were gathered. However, the papers which only have abstracts were excluded because of insufficient data for meta-analysis. Therefore, we first read the titles of the publications and the abstracts to find exactly those articles that examined the relationship between p-AKT and overall survival in patients with NSCLC. After the abstracts met these conditions, the full texts were analyzed and included into our meta-analysis according to the following criteria: (1) studies were written as full paper; (2) expression levels of p-AKT were compared to patient’s overall survival; (3) expression of the proteins were evaluated in tumor tissues by immunohistochemistry (IHC) or reverse transcription and polymerase chain reaction (RT-PCR) analysis; (4) Hazard ratios (HR) and 95% CI for overall survival were provided or could be calculated from the sufficient data; (5) if the same group of patients were used to analyze more than once, the most complete research was selected for our study.

### Data Extraction

 Two reviewers (Qiu Zhi-Xin and Kui Zhang) independently checked all articles and extracted data in separate databases. The following information were collected from each study: first author’s name, year of publication, ethnicity, sample size, laboratory methodology, cut-off value, smoking status, histological type, clinical stage and HR with 95% CI. Disagreements were resolved through discussion among the authors.

### Statistical Analysis

 The intensity of relationship between the expression levels of p-AKT and overall survival were described as HRs. Positive expression of p-AKT indicated poor prognosis in patients with NSCLC if HR>1 with the 95% CI did not overlap 1. From some published researches, HR and 95% CI could be directly obtained by using univariate or multivariate survival analysis. Otherwise, HR and 95% CI were calculated by Kaplan-Meier survival curves using the software Engauge Digitizer Version 4.1 (http://digitizer.sourceforge.net/) and the method presented by Parmar et al. before [13,14]. Then, extracted data were utilized to reconstruct the HR and its variance (GraphPad Software, Inc, La Jolla, CA, USA).

 The pooled HR corresponding to the 95% CI was used to assess the prognostic value of AKT or p-AKT in patients. Statistical heterogeneity was tested by Cochrane’s Q test (Chi-squared test; Chi^2^) and inconsistency (I^2^) [15,16]. If there was no obvious heterogeneity, the fixed-effects model (Mantel-Haenszel method) was used to estimate the pooled HR; otherwise, the random-effects model (DerSimonian and Laid method) was used [13]. Funnel plot and Begg’s rank correlation method were designed for assessing risk of publication bias. STATA 12.0 (STATA Corp., College, TX) was used to perform statistical analysis.

## Results

### Study Selection and Characteristics

**Figure 1:**
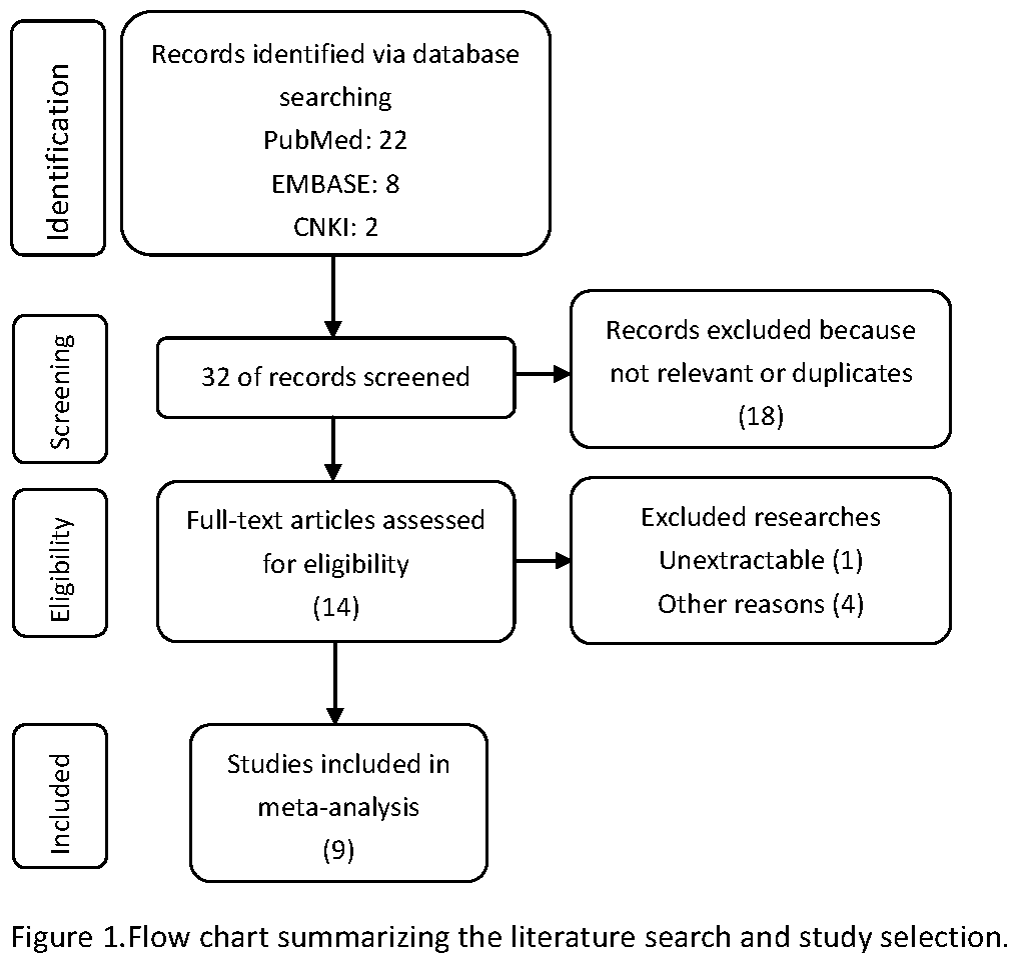
Flow chart summarizing the literature search and study selection.

 22, eight and two articles were retrieved from PubMed, EMBASE and CNKI electronic database according to our defined keywords and text words, respectively (Figure *1*). Then, via careful reading the abstracts, 14 researches that focused on the association between the expression of p-AKT and survival were included in our full-text review process. After reading the full-text researches, 5 papers had to be excluded because data were not extractable or could not provide enough information about overall survival. Finally 9 studies including 1049 cases were available for our meta-analysis. Among all the included studies, 7 papers were in English and 2 papers were Chinese.

 The individual characteristic of the eligible researches are summarized in Table 1, 5 studies included patients from Asia, 2 from Europe and 2 from America, respectively. Expressions of p-AKT were all detected via IHC. According to univariate analysis, 3 studies provided the HR with 95% CI directly, 6 studies showed survival curves that available to calculate the HR. However, 4 studies provided the HR with 95% CI directly, 1 study showed survival curves that available to calculate the HR, the other 4 papers had no data available by multivariate analysis.

**Table 1:** Main characteristics and results of eligible studies.

First Author	Year	Ethnicity	Cases	P/N	Method	Cutoff value	Smoking status	Histological type	Stage	Overall Survival					
							**(Yes/No)**	**(SCC/ADC/**	**(Ⅰ/Ⅱ/**	**Univariate**			**Univariate**		
								**Others)**	**Ⅲ/Ⅳ)**	**HR Estimate**	**HR**	**95% CI**	**HR Estimate**	**HR**	**95% CI**
Odile David	2004	America	61	14/47	IHC	>0%	NA	NA	NA	Sur. Curve	2.65	2.19-3.30	NA	NA	NA
Cappuzzo F.	2004	Italian	103	51/52	IHC	≥2 scores	84/19	21/57/25	0/0/ 14/89	Sur. Curve	0.84	0.56-0.98	HR 95%CI	0.58	0.35-0.94
Amit Shah	2005	Irishman	82	41/41	IHC	>0%	NA	46/22/14	47/18/ 17/0	Sur. Curve	0.43	0.27-0.69	HR 95%CI	0.36	0.18-0.71
Junji Tsurutani	2007	Japanese	46	44/2	IHC	>0%	37/9	37/8/1	16/11/ 8/9	HR 95%CI	1.06	0.54-2.06	Sur. Curve	0.89	0.64-1.24
Akihiko Yoshizawa	2010	America	265	209/56	IHC	＞TS2	NA	128/137/0	NA	HR 95%CI	2.02	1.34-3.19	HR 95%CI	1.83	1.11-3.22
Pu Rong	2010	Chinese	137	81/56	IHC	>2 scores	NA	61/76/0	13/60/ 47/17	Sur. Curve	2.78	1.18-4.67	NA	NA	NA
Dan Liu	2011	Chinese	158	118/40	IHC	>2 scores	NA	75/77/20	NA	HR 95%CI	1.06	0.54-2.06	NA	NA	NA
ChenYao-hua	2011	Chinese	103	58/45	IHC	>0%	62/41	62/34/7	16/40/ 35/12	Sur. Curve	1.79	1.55-2.13	NA	NA	NA
She-Juan An	2012	Chinese	95	43/52	IHC	＞0 scores	60/38	24/62/12	47/14/ 27/10	Sur. Curve	3.54	1.63-5.94	HR 95%CI	3.384	1.737-6.593

Abbreviation: P/N, positive expression/negative expression; IHC, immunohistochemistry; SCC, squamous cell carcinoma; ADC, adenocarcinoma; HR, hazard ratio; NA, no available or no applicable.

### Meta-analysis

 We evaluated whether p-AKT expression levels were associated with the overall survival in patients with NSCLC. Of the 9 trials evaluable for systematic review, 4 could not be included in meta-analysis by multivariate analysis due to insufficient data to estimate the HR and 95% CI.

**Figure 2:**
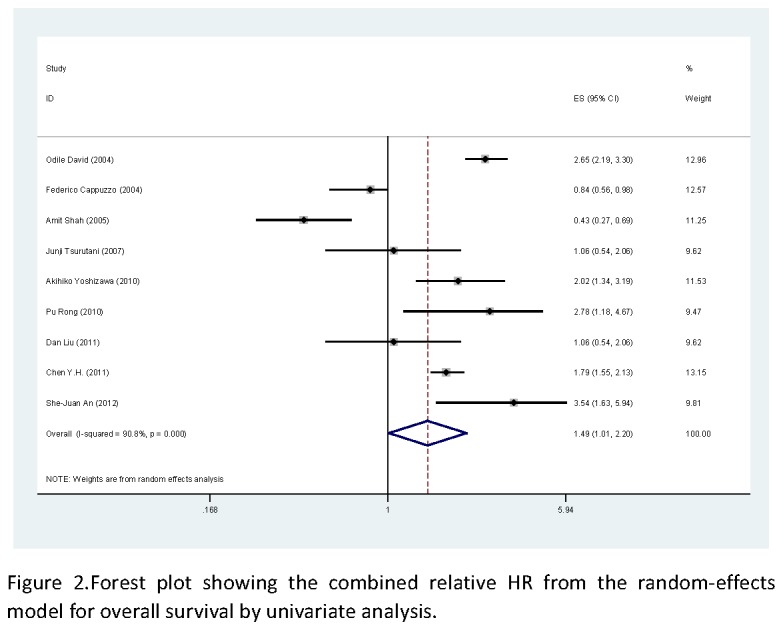
Forest plot showing the combined relative HR from the random-effects model for overall survival by univariate analysis.

**Figure 3:**
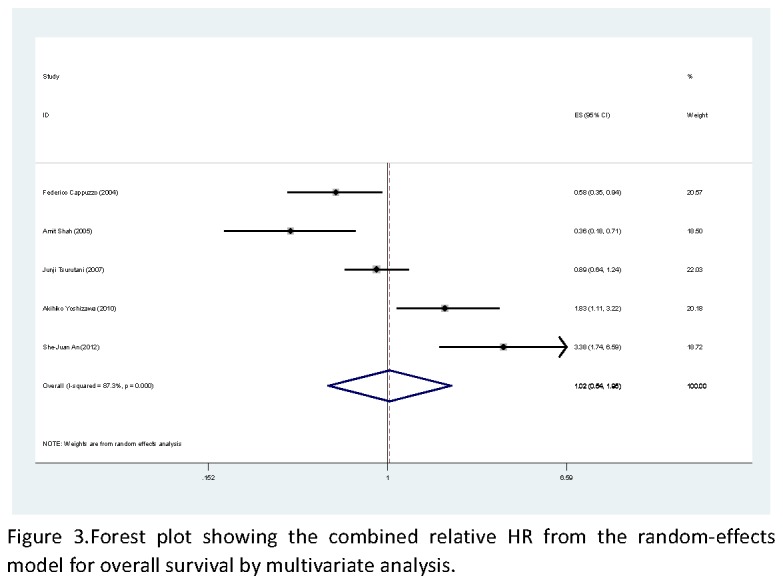
Forest plot showing the combined relative HR from the random-effects model for overall survival by multivariate analysis.

 Nine studies, including 1049 patients, reported the effect of p-AKT on overall survival using analyses unadjusted for other factors [17-24]. As shown in Figure *2*, p-AKT was significantly correlated with worse overall survival according to univariate analysis, with a combined HR of 1.49 (95% CI: 1.01-2.20, *P*<0.05). The random-effects model (the DerSimonian and Laird method) was used because of significant heterogeneity was observed among these researches (*P*=0.000, I^2^=90.8%). Five studies, demonstrated the effect of p-AKT on overall survival using analyses adjusted for other factors, including 590 patients [18-21,24]. As shown in Figure *3*, no statistically significant was observed between the expression of p-AKT levels and overall survival, with a combined HR of 1.02 (95% CI: 0.54-1.95). The random-effects model (the DerSimonian and Laird method) was used because of significant heterogeneity was observed among these researches (*P*=0.000, I^2^=87.3%).

Next, we performed subgroup analyses to investigate if there were differences in results with respect to the ethnicity, year of publication and cut off values in which the study was conducted. However, because of the limited number of studies that we couldn’t get the statistically significant results mostly (*Table S1 and* Table *S2*). Thus, more studies should be conducted in the future.

**Figure 4:**
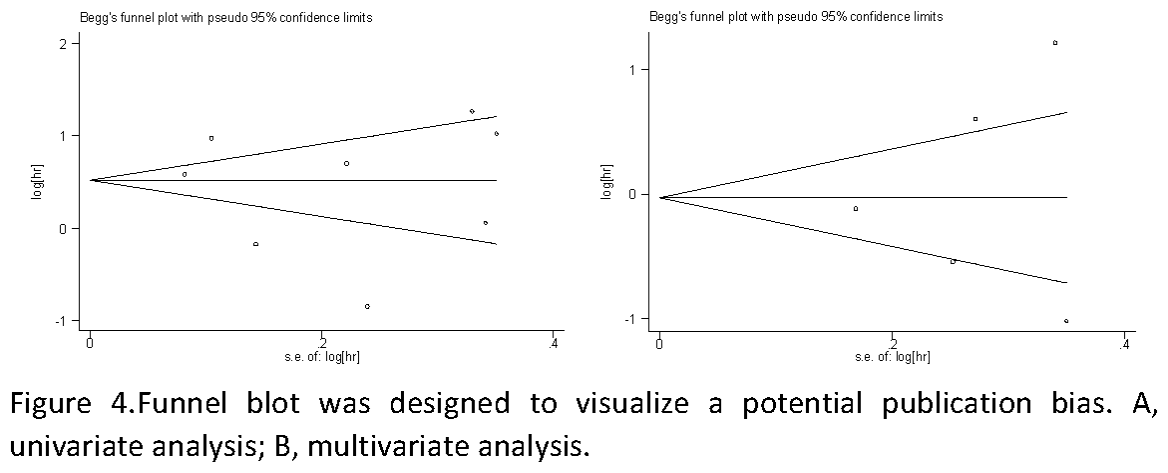
Funnel blot was designed to visualize a potential publication bias.

 Publication bias statistics were determined using the method of Begg’s test (Figure *4*). No publication biases were found in the nine overall survival studies used for univariate analysis (*P*=0.509) and five overall survival studies used for multivariate analysis (*P*=0.786) . Sensitively analysis was performed to investigate the effect of every study on the overall meta-analysis by omitting one study each time, and the omission of any study made no significant difference, demonstrating that our results were statistically reliable.

## Discussion

 The prognostic role of a specific molecular marker is more powerful when used to help make therapeutic decision. In earlier studies, various kinds of genetic alterations have been identified as prognostic factors such as HER-2 in breast carcinoma and EGFR gene in NSCLC. However, most other clinically useful molecular markers which have predictive value of the therapeutic response and prognostic value failed to demonstrate usefulness in subsequent investigations. Because of a great deal of controversy results of the prognostic implications of p-AKT in NSCLC remains, we undertook a meta-analysis to determine whether p-AKT can serve as a prognostic marker for patients with NSCLC.

 Our meta-analysis focuses on p-AKT expression in resected NSCLC. We also have collected the information about the relationship between the p-AKT expression and progression-free survival at the same time, but we have not conducted the meta-analysis for it because of insufficient data could be used. Although the results of two studies [18,19] which reported the impact of p-AKT expression on overall survival have the opposite conclusion compared with the other five studies [17,21,22,24] in the meta-analysis, our meta-analysis with accumulated data suggested that overexpression of p-AKT was associated with shorter overall survival time and predicted worse prognosis in patients with NSCLC. The pooled hazard ratio (HR) for overall survival was 1.49 (95% confidence interval (CI): 1.01-2.20) by univariate analysis and 1.02 (95% CI: 0.54-1.95) by multivariate analysis.

 AKT has been found to play a role in the survival of cancer cells in breast, prostate, ovary, and brain tissue [25-28]. It was also identified as a constitutively active kinase that promotes survival of NSCLC cell [29]. Recently, a clinical study of p-AKT expression in tissue from patients with bronchial epithelial dysplasia and malignancy corroborates this hypothesis. They observed strong p-AKT staining in 12 of 44 normal bronchial biopsy specimens, 4 of 9 reactive specimens, 22 of 25 dysplastic specimens, and 25 of 76 NSCLC specimens. Tsao et al. [30] considered that AKT activation may be more strongly associated with the early stages of malignant transformation than with progression to frank neoplasia [17]. 

Moreover, the prognostic significance of p-AKT expression has been examined in many cancers, although evidence is still limited. Ermoian et al. [6] found no association between p-AKT and patient survival in gliomas; but the expression of PTEN significantly prognosis in this patients. In renal cell carcinoma, p-AKT was associated with poor prognosis on univariate but no multivariate analysis [31]. However, in breast cancer, p-AKT does not seem to predict survival but does predict patient relapse [32,33]. Thus, evidences suggest that p-AKT in other tumor types might be associated with factors that predict worse prognosis, such as histology, tumor stage, grade, metastatic disease. In NSCLC, the results of prognostic value of p-AKT are not consistent and have not been well summarized.

Our meta-analysis is based on published data and was performed using univariate analysis followed by further multivariate analysis, which is the first time to evaluate the effect of p-AKT on overall survival. Some limitations exist in our study. The risks calculated in our meta-analysis may be an overestimate due to publication and reporting bias. Positive results tend to be accepted by journals, whereas negative results often are rejected or even not be submitted. Furthermore, we did not include unpublished papers and abstracts into meta-analysis because the required data was available only in full publications. Another potential source of bias is related to the method used to extrapolate the HR. HR was extracted from the data included in the article directly or calculated from the survival curves. Actually, the method of extrapolating HR from survival curves seems to be less reliable because this strategy did not completely eliminate inaccuracy in the extracted survival rates. Moreover, we also think that different therapy strategies in these studies have different impact on overall survival, so this factor should be taken into consideration. Unfortunately, only one of these studies has described the therapy strategy after the patients have diagnosed the lung cancer. These patients received gefitinib daily at a dose of 250 mg. Another one research has just mentioned all the patients received no neoadjuvant therapy before they have surgical resection. Therefore, more meticulous research should be conducted. Additionally, different antibodies might have an influence on the study, so we summarized the information of antibody sources in the Table *S3*. The antibodies of p-AKT in five studies were bought from “Cell Signaling Technology, Beverly, MA”, in the other three studies were bought from different companies, and only one study didn’t give the antibody source information. Then we did the stratified analysis, because of the limited number of studies that the results have no statistically significant. Nevertheless, no publication bias was detected using Begg’s test (P>0.05), indicating that the statistics obtained approximate the actual results. Sensitivity analysis was also conducted to investigate the influence of a single study on the overall meta-analysis by omitting one study at a time, and the omission of any study made no significant difference, suggesting that our results were statistically reliable.

In summary, overexpression of p-AKT was associated with poor overall survival in patients with NSCLC on univariate analysis but not multivariate analysis. However, more prospective clinical studies are needed to explore the prognostic value of p-AKT expression in NSCLC.

## Supporting Information

Checklist S1 **PRISMA checklist.**
(DOC)

Table S1 **Subgroup analyses by univariate analysis.**
(DOCX)

Table S2 **Subgroup analyses by multivariate analysis.**
(DOCX)

Table S3 **Information of antibody sources.**
(DOCX)
