# Supplementary material for: The Prognostic Value of Phosphorylated AKT Expression in Non-Small Cell Lung Cancer: A Meta-Analysis
Source: PLoS One. 2013 Dec 5;8(12):e81451. doi: 10.1371/journal.pone.0081451 (PMC3857807; doi:10.1371/journal.pone.0081451)
Supplement: Table S1 — Subgroup analyses by univariate analysis. (DOCX) [file pone.0081451.s002.docx]

Table S1 Subgroup analyses by univariate analysis

| Subgroup |  | **Pooled Data(Random)** | | | **Test for Heterogeneity** | |
| --- | --- | --- | --- | --- | --- | --- |
|  |  | **No. of Studies** | **OR** | **95%CI** | ***P*-value** | **I^2^(%)** |
| Year | 2004 | 2 | 1.17 | 0.60-2.27 | 0.002 | 89.3 |
|  | 2005 | 1 | 2.33 | 1.40-3.72 | NA | NA |
|  | 2007 | 1 | 1.06 | 0.54-2.07 | NA | NA |
|  | 2010 | 2 | 1.89 | 1.40-2.54 | 0.677 | 0.0 |
|  | 2011 | 2 | 0.84 | 0.61-1.18 | 0.448 | 0.0 |
|  | 2012 | 1 | 2.54 | 1.63-3.95 | NA | NA |
| Ethnicity | America | 2 | 1.78 | 1.37-2.31 | 0.467 | 0.0 |
|  | Italian | 1 | 0.84 | 0.63-1.11 | NA | NA |
|  | Irishman | 1 | 2.33 | 1.46-3.72 | NA | NA |
|  | Japanese | 1 | 1.06 | 0.54-2.07 | NA | NA |
|  | Chinese | 4 | 1.40 | 0.80-2.46 | 0.000 | 83.9 |
| Cutoff value | ＞level2 | 1 | 1.65 | 1.19-2.29 | NA | NA |
|  | ≥2 scores | 1 | 0.84 | 0.63-1.11 | NA | NA |
|  | ＞0% | 3 | 1.25 | 0.61-2.54 | 0.002 | 84.6 |
|  | ＞TS2 | 1 | 2.02 | 1.31-3.12 | NA | NA |
|  | ＞2 scores | 2 | 1.47 | 0.90-2.40 | 0.195 | 40.4 |
|  | ＞0 scores | 1 | 2.54 | 1.63-3.95 | NA | NA |

NA, no available or no applicable.
