# Supplementary material for: The Prognostic Value of Phosphorylated AKT Expression in Non-Small Cell Lung Cancer: A Meta-Analysis
Source: PLoS One. 2013 Dec 5;8(12):e81451. doi: 10.1371/journal.pone.0081451 (PMC3857807; doi:10.1371/journal.pone.0081451)
Supplement: Table S2 — Subgroup analyses by multivariate analysis. (DOCX) [file pone.0081451.s003.docx]

Table S2 Subgroup analyses by multivariate analysis

| Subgroup |  | **Pooled Data(Random)** | | | **Test for Heterogeneity** | |
| --- | --- | --- | --- | --- | --- | --- |
|  |  | **No. of Studies** | **OR** | **95%CI** | ***P*-value** | **I^2^(%)** |
| Year | 2004 | 1 | 0.58 | 0.35-0.95 | NA | N |
|  | 2005 | 1 | 2.81 | 1.40-5.65 | NA | NA |
|  | 2007 | 1 | 0.89 | 0.64-1.24 | NA | NA |
|  | 2010 | 1 | 1.83 | 1.07-3.12 | NA | NA |
|  | 2012 | 1 | 3.38 | 1.74-6.59 | NA | NA |
| Ethnicity | America | 1 | 1.83 | 1.07-3.12 | NA | NA |
|  | Italian | 1 | 0.58 | 0.35-0.95 | NA | NA |
|  | Irishman | 1 | 2.81 | 1.40-5.65 | NA | NA |
|  | Japanese | 1 | 0.89 | 0.64-1.24 | NA | NA |
|  | Chinese | 1 | 3.38 | 1.74-6.59 | NA | NA |
| Cutoff value | ≥2 scores | 1 | 0.58 | 0.35-0.95 | NA | NA |
|  | ＞0% | 2 | 1.52 | 0.49-4.66 | 0.004 | 88.3 |
|  | ＞TS2 | 1 | 1.83 | 1.07-3.12 | NA | NA |
|  | ＞0 scores | 1 | 3.38 | 1.74-6.59 | NA | NA |

NA, no available or no applicable.
