# Supplementary material for: The Prognostic Value of Phosphorylated AKT Expression in Non-Small Cell Lung Cancer: A Meta-Analysis
Source: PLoS One. 2013 Dec 5;8(12):e81451. doi: 10.1371/journal.pone.0081451 (PMC3857807; doi:10.1371/journal.pone.0081451)
Supplement: Table S3 — Information of antibody sources. (DOCX) [file pone.0081451.s004.docx]

Table 3 Information of antibody sources

| **First Author** | **Antibody Sources** | **PFS** | **Treatment strategies** |
| --- | --- | --- | --- |
| Odile David | Cell Signaling Technology, Beverly, MA | NA | NA |
| Cappuzzo F. | Cell Signaling Technology, Beverly, MA | NA | Received gefitinib daily at a dose of 250 mg. Patients received the drug until their disease progressed (n=77), they experienced unacceptable toxicity (n=1), or they refused to comply further (n=1). |
| Amit Shah | New England Biolabs | NA | NA |
| Junji Tsurutani | NA | NA | NA |
| Akihiko Yoshizawa | Cell Signaling Technology, Beverly, MA | NA | No patients received neoadjuvant therapy. |
| Pu Rong | SAB Company | NA | NA |
| Dan Liu | Cell Signaling Technology, Beverly, MA | NA | NA |
| Chen Yao-hua. | Santa Cruz  Biotechnology Company | NA | NA |
| She-Juan An | Cell Signaling Technology, Beverly, MA | NA | NA |

NA, no available or no applicable.
